# Health Care Utilization Profiles in Young Ukrainian Refugee Children

**DOI:** 10.1001/jamanetworkopen.2026.26021

**Published:** 2026-07-29

**Authors:** Bartosz Dobies, Konrad Pędziwiatr, Iwona A. Bielska

**Affiliations:** 1 Institute of Public Health, Jagiellonian University, Krakow, Poland; 2 Doctoral School of Medical and Health Sciences, Jagiellonian University, Krakow, Poland; 3 Center for Advanced Studies of Population and Religion, Krakow University of Economics, Krakow, Poland; 4 Department of Health Research Methods, Evidence and Impact, McMaster University, Hamilton, Ontario, Canada

## Abstract

**Importance:**

Understanding how Ukrainian refugee children accessed the health care system after fleeing the war is essential to inform future preparedness and resource allocation in host countries.

**Objective:**

To use latent class analysis of registry data to identify distinct health care utilization profiles among young Ukrainian refugee children who accessed the health care system in southern Poland in 2022.

**Design, Setting, and Participants:**

This registry-based retrospective cohort study used electronic health record data from 2022 and included Ukrainian refugee children, aged 0 to 5 years, who received health care services in facilities in Małopolska Voivodeship, Poland. Participants were followed up from February 24 through December 31, 2022. The data were analyzed from April to October 2025.

**Exposure:**

Healthcare utilization indicators among postwar displaced Ukrainian refugee children used as inputs to the latent profile analysis.

**Main Outcomes and Measures:**

Latent profile membership of health care utilization based on health care visit types, *International Statistical Classification of Diseases and Related Health Problems, Tenth Revision (ICD-10)* coded diagnoses, and intensity of health care use, with temporal patterns of system entry and service trajectories examined after profile identification.

**Results:**

The analytic sample included 9845 Ukrainian refugee children aged 0 to 5 years (4849 [49.3%] female) who received 35 199 health care services in Małopolska Voivodeship in 2022. Age at first health care contact was evenly distributed across categories (mean [SD] of 2.7 [1.6] years), 3802 children (38.6%) had a single recorded service, 1845 (18.7%) had more than 5 services, and nearly half (4505 [45.8%]) received care in the regional capital (Krakow). Based on latent class analysis, 5 pediatric patient profiles were identified: mostly primary care (5216 [53.0%]), hospitalized with infectious diseases (1539 [15.6%]), highest health care use (1329 [13.5%]), emergency care for injuries (900 [9.1%]), and dental and preventive care (861 [8.7%]), differing in visit type, diagnostic patterns, and health care utilization intensity. Temporal patterns varied across profiles with earlier system entry among children requiring hospitalization for infectious diseases or emergency care for injuries, and later entry among those using primary care or dental and preventive care.

**Conclusions and Relevance:**

In this cohort study of pediatric Ukrainian refugees in southern Poland, distinct health care utilization patterns were observed with early reliance on hospital and emergency care followed by greater use of primary services. These findings underscore the need for refugee-hosting countries to rapidly adapt health care resources, prioritizing inpatient and emergency care in the initial months following a crisis.

## Key Points

**Question:**

What distinct latent profiles of short-term health care needs were identified among Ukrainian refugee children, aged 5 years and under, who used the health care system in Southern Poland in 2022?

**Findings:**

This cohort study identified 5 distinct health care utilization profiles among 9845 Ukrainian refugee children, ranging from low-intensity primary care to intensive hospital and emergency use, with earlier health care system entry among children requiring hospitalization for infectious diseases or emergency care for injuries.

**Meaning:**

These findings suggest that refugee-hosting countries must be prepared to rapidly adapt health care resources, prioritizing emergency and inpatient care in the initial months following a crisis.

## Teaser

This cohort study uses registry data to analyze health care utilization profiles among young Ukrainian refugee children who accessed the health care system in southern Poland in 2022.

## Introduction

The onset of the full-scale invasion of Ukraine by Russia in 2022 led to the forced displacement of nearly 4 million people to the European Union that year.^1^ Approximately 1 million of these forcibly displaced migrants, of whom 43% were children, settled in neighboring Poland (population: 38 million).^2,3^ There, they received Temporary Protection and identification numbers granting primary, outpatient, and inpatient health coverage akin to that of other insured residents.^4^ A substantial area of refugee settlement was southern Poland due to its proximity to Ukraine (approximately 200 km), as well as highway and rail connections, which served as evacuation routes.^5^

Previous studies show that pediatric refugees are at heightened risk for illness due to exposure to long travel times, group living conditions, and poor nutrition.^5,6,7^ Their care often requires culturally specific approaches and understanding of the prevalence of diseases in the countries of origin.^8,9,10^ Additional challenges to delivering care include language and system navigation barriers.^11,12^ Based on published reviews, young refugee children (up to age 6 years) who relocated to high-income countries were diagnosed with nutritional deficiencies, respiratory infections, gastrointestinal illness, and neurodevelopmental disorders.^9,13^

Research from Poland indicates that during the initial weeks of the refugee influx, forcibly displaced Ukrainian children most often sought care for communicable conditions and chronic disease management.^14,15^ Hospitalizations up to June 2022 were highest among children aged 0 to 5 years.^16^ Cases of tuberculosis, viral hepatitis, and HIV were noted, requiring swift connection to specialized therapies.^10,15^ Additionally, Poland provided treatment for medically evacuated pediatric patients, such as those with neoplasms.^17^ Although studies on the health of Ukrainian refugees were conducted in Poland, they were based on data from single medical sites or refugee reception points,^14,15,18,19^ analyzed data collected over short periods following the initial response,^14,16^ or were of limited scope, focusing on specific conditions.^10,16,20,21^ As such, comprehensive knowledge on the status of the pediatric population is lacking.

During emergencies, such as large-scale forced migration, knowledge of the health status of the refugee population is important to prepare health systems for preventing the spread of infectious disease, determining adequate levels of care, and providing medical follow-up for chronic diseases.^22,23,24^ Establishing the needs of young refugee children is crucial, as those less than age 5 years are especially vulnerable to experiencing negative clinical outcomes when faced with infectious diseases, including higher mortality risk.^25^ Therefore, the objective of this study was to use a data-driven approach to registry information to identify distinct latent patient profiles of Ukrainian refugee children, aged 5 years and younger, who accessed the health care system in Poland in 2022.

## Methods

### Study Sample

This retrospective cohort study looked at a population of children, 5 years or younger, who received health care services at facilities in Małopolska Voivodeship, Poland, and had health care coverage as displaced persons as per the Act on Assistance to Citizens of Ukraine in Connection With the Armed Conflict on the Territory of That State or had a comorbid *International Statistical Classification of Diseases and Related Health Problems, Tenth Revision (ICD-10)* Z65.5 diagnosis (“exposure to disaster, war and other hostilities”).^4^ Data on all publicly covered health care encounters between February 24 and December 31, 2022, were obtained from the Małopolska Voivodeship branch of the National Health Fund (Narodowy Fundusz Zdrowia)^5^ and analyzed from April to October 2025. Małopolska Voivodeship is located in southern Poland (total population size 3.4 million; largest city, Kraków)^26^ and was a major region of reception of Ukrainian refugees.^5^ In 2022, 37 207 forced migrant children from Ukraine, including 10 573 aged 5 years and under, were registered there.^3^ The study was approved by the University Ethics Committee for Scientific Research at Kraków University of Economics and informed consent was waived for the secondary analysis of deidentified administrative data for research purposes in accordance with general data protection regulation. This study followed the Strengthening the Reporting of Observational Studies in Epidemiology (STROBE) reporting guidelines for cohort studies.

### Measures

The routinely collected dataset included information on the timing and setting of health care services, patient diagnoses, and the main *ICD-10* diagnostic code assigned to each health care visit. Demographic data included age and gender. Records captured services in primary care, emergency department (ED) care, hospital care (inpatient or outpatient), dentistry, specialist outpatient care, psychiatry (inpatient or outpatient), medical rehabilitation (inpatient or outpatient), palliative and hospice care (inpatient or outpatient), and services provided under separate contracts. Categories with low utilization (dentistry, specialist outpatient care, psychiatry, rehabilitation, palliative and hospice care, separately contracted services) were combined into an other category.

To quantify overall service use, an intensity index was calculated for each individual as their total number of health care services in 2022 divided by the length of the observation period, defined as the number of months between the first and last recorded service (including both months). For the main analysis, the dataset was transformed into binary variables reflecting health care utilization and diagnostic characteristics. Patients were classified as having greater service use if they had more than 5 services in 2022. Additional binary indicators included having an intensity index of 2 or greater, at least 1 hospital visit, at least 1 ED visit, at least one visit to other services, and whether more than 50% of the visits occurred in primary care settings. Diagnoses were dichotomized so that the presence of any *ICD-10* code indicated at least 1 documented diagnosis. *ICD-10* codes were grouped by *ICD-10* blocks and reduced to the most frequent categories in the sample with the remaining codes combined into an other category.

### Statistical Analysis

Latent class analysis (LCA) is used to uncover distinct subgroups within a population, even when individuals appear similar based on observable traits, assuming that membership in hidden groups can be inferred from response patterns.^27^ LCA has been applied to define complex patient profiles by grouping individuals with high health care utilization into clinically meaningful subtypes.^28^ In this study, LCA was conducted to identify data-driven patient profiles based on diagnostic codes, visit frequency, and care settings. The analysis was performed using the poLCA package in R version 1.6.0.1 (R Project for Statistical Computing) with a maximum of 5000 iterations and 10 repetitions using random starting values to avoid local maxima.

Models with an increasing number of classes (ranging from 2-10) were estimated. Model fit for each setting was assessed using the Akaike information criterion, bayesian information criterion, entropy, log-likelihood, likelihood ratio test, and estimated class sizes. The aim was to identify the most parsimonious and interpretable solution. The robustness of the selected latent class solution was assessed through stability analysis using 100 random subsamples, each comprising 80% of the dataset. Agreement between the original and subsampled class assignments was assessed using the Adjusted Rand Index. To evaluate potential redundancy or violation of the local independence assumption, the Jaccard similarity index was computed to assess the pairwise similarity between binary variables. Values greater than 0.4 were used as a threshold to exclude 1 variable from each highly similar pair.

Patients’ characteristics were summarized using frequencies and proportions for categorical variables, and medians with first and third quartiles for continuous variables. Comparisons between groups were conducted using Pearson χ^2^ test for categorical variables and the Kruskal-Wallis rank sum test for continuous variables. Statistical tests were 2-sided with a significance level of α = .05. All analyses were conducted in R version 4.4.3 (R Project for Statistical Computing).

## Results

### Participants

The study included 9845 pediatric Ukrainian refugees (4849 [49.3%] female; 4990 [50.7%] male) who received 35 199 health care services in Małopolska Voivodeship in 2022 with age at first health care visit being fairly evenly distributed across categories (mean [SD] of 2.7 [1.6] years). For health care utilization, 3802 (38.6%) had only 1 recorded service, whereas 1845 (18.7%) had more than 5. The intensity index was greater than 1 for 4210 children (42.8%) and less than or equal to 1 for the remaining 5635 (57.2%) (Table 1).

**Table 1.** Characteristics of Pediatric Ukrainian Refugee Patients Aged 0 to 5 Years in Małopolska, Poland

Characteristic	Participants, No (%)						*P* value
	Total	Pediatric Ukrainian refugee patients’ profiles					
		Mostly primary care	Hospitalized with infectious diseases	Highest health care use	Emergency care for injuries	Dental and preventive care	
No. (%)	9845 (100.0)	5216 (53.0)	1539 (15.6)	1329 (13.5)	900 (9.1)	861 (8.7)	NA
Age at first visit, y							
< 1	1171 (11.9)	513 (9.8)	277 (18.0)	192 (14.4)	31 (3.4)	158 (18.4)	<.001^a^
1	1720 (17.5)	1017 (19.5)	238 (15.5)	256 (19.3)	130 (14.4)	79 (9.2)	
2	1721 (17.5)	975 (18.7)	294 (19.1)	202 (15.2)	178 (19.8)	72 (8.4)	
3	1715 (17.4)	905 (17.4)	261 (17.0)	226 (17.0)	195 (21.7)	128 (14.9)	
4	1763 (17.9)	931 (17.8)	235 (15.3)	217 (16.3)	201 (22.3)	179 (20.8)	
5	1755 (17.8)	875 (16.8)	234 (15.2)	236 (17.8)	165 (18.3)	245 (28.5)	
Gender							
Female	4849 (49.3)	2603 (49.9)	758 (49.3)	621 (46.7)	442 (49.1)	425 (49.4)	.35^a^
Male	4990 (50.7)	2607 (50.0)	781 (50.7)	708 (53.3)	458 (50.9)	436 (50.6)	
Not specified	6 (<0.1)	6 (0.1)	0	0	0	0	
No. of medical services							
1	3802 (38.6)	2427 (46.5)	703 (45.7)	0	486 (54.0)	186 (21.6)	<.001^a^
2-3	2811 (28.6)	1729 (33.1)	564 (36.6)	0	242 (26.9)	276 (32.1)	
4-5	1387 (14.1)	704 (13.5)	272 (17.7)	7 (0.5)	168 (18.7)	236 (27.4)	
>5	1845 (18.7)	356 (6.8)	0	1322 (99.5)	4 (0.4)	163 (18.9)	
Intensity index							
<1	1163 (11.8)	640 (12.3)	152 (9.9)	134 (10.1)	111 (12.3)	126 (14.6)	<.001^a^
1	4472 (45.4)	2823 (54.1)	759 (49.3)	88 (6.6)	530 (58.9)	272 (31.6)	
>1	4210 (42.8)	1753 (33.6)	628 (40.8)	1107 (83.3)	259 (28.8)	463 (53.8)	
Mo. of health care system entry, median (IQR), mo	5.00 (4.00-7.00)	5.00 (4.00-8.00)	4.00 (3.00-7.00)	5.00 (4.00-6.00)	4.00 (3.00-7.00)	6.00 (4.00-8.00)	<.001^b^
Entered the health care system between February and April	3981 (40.4)	1891 (36.3)	774 (50.3)	587 (44.2)	458 (50.9)	271 (31.5)	<.001^a^
Time between first and last recorded visit, median	1.00 (1.00-4.00)	1.00 (1.00-2.00)	1.00 (1.00-1.00)	6.00 (4.00-8.00)	1.00 (1.00-2.00)	2.00 (1.00-4.00)	<.001^b^
Received care in regional capital (Kraków)	4505 (45.8)	1872 (35.9)	977 (63.5)	614 (46.2)	650 (72.2)	392 (45.5)	<.001^a^

Abbreviation: NA, not applicable.

^a^ Analyzed with Pearson χ^2^ test.

^b^ Analyzed with Kruskal-Wallis rank sum test.

**Figure 1.**
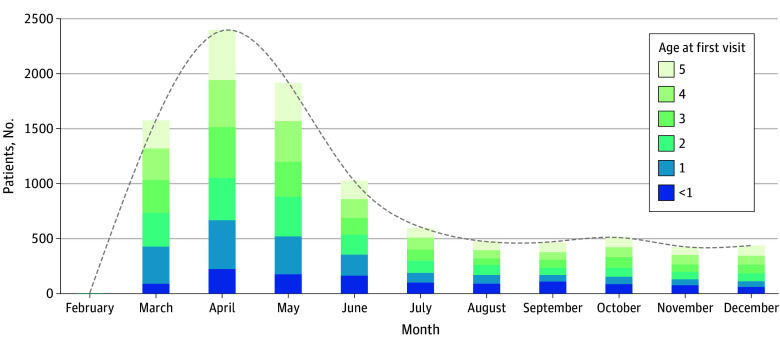
Bar Chart of Monthly New Pediatric Ukrainian Refugee Patients Aged 0 to 5 Years Entering the Health Care System in 2022 (N = 9845) A smoothed line represents a generalized additive model fit to total patient counts per month.

The age distribution remained stable across months, suggesting a consistent demographic composition (Figure 1). A clear peak in health care system entry occurred in April, shortly after the full-scale Russian invasion, followed by a gradual decline. The median month of first health care contact was May with 3981 children (40.4%) entering between February and April. Nearly half of the cohort (4505 [45.8%]) received health care services in the regional capital city of Kraków (Table 1).

### Co-Occurrence of Health Care Utilization Patterns

A matrix of Jaccard similarity indices illustrating co-occurrence patterns between health care utilization metrics and *ICD-10*-coded disease categories is shown in eFigure 1 in Supplement 1. Greater visit intensity (>5 visits) was associated with having a diagnosis of factors influencing health status (*ICD-10*, Z00-Z99; Jaccard similarity index, 0.32), at least 1 other visit (Jaccard similarity index, 0.31), abnormal clinical and laboratory findings (*ICD-10*, R00-R99; Jaccard similarity index, 0.26), and respiratory diseases (*ICD-10*, J00-J99; Jaccard similarity index, 0.26). Certain visit types showed associations with specific *ICD-10* blocks: hospital visits with infectious and parasitic diseases (*ICD-10*, A00-A99; Jaccard similarity index, 0.25), ED visits with injuries and poisonings (*ICD-10*, S00-S99; Jaccard similarity index, 0.29), and other visits with digestive system diagnoses (*ICD-10*, K00-K93; Jaccard similarity index, 0.28), primarily involving dental care.

A high degree of similarity was seen between individuals with more than half of their visits in primary care and those with diseases of the respiratory system (*ICD-10*, J00-J99; Jaccard similarity index, 0.58) or with factors influencing health status (*ICD-10*, Z00-Z99; Jaccard similarity index, 0.41). Consequently, *ICD-10* blocks J00-J99 and Z00-Z99 were excluded from the LCA due to their high Jaccard similarity with frequent primary care visits, indicating potential redundancy and a risk of violating the local independence assumption.

### Latent Class Model Selection

The optimal solution identified 5 latent classes. This model had the highest entropy, indicating clearer class separation and improved interpretability. Both the Akaike information criterion and bayesian information criterion showed an elbow at 5 classes (eFigure 2 in Supplement 1), after which the rate of decrease in these values slowed substantially. Furthermore, models with 6 or more classes began to include smaller subgroups representing fewer than 5% (n < 493) of the population (Table 2), limiting the practical interpretability of those additional classes. The latent class solution demonstrated excellent stability in subsampling analyses with a mean (SD) Adjusted Rand Index of 0.989 (0.013), indicating near-perfect agreement in class assignments across random subsamples (eFigure 3 in Supplement 1).

**Table 2.** Selection Criteria for Latent Class Analysis (N = 9845)

Class	AIC	BIC	Entropy	Log-likelihood	LRT	Class sizes, %
2	110 573	110 810	0.817	−55 253	<0.001	1, 52.4; 2, 47.6
3	106 847	107 207	0.881	−53 373	<0.001	1, 56.4; 2, 27.5; 3, 16.1
4	104 637	105 119	0.901	−52 251	<0.001	1, 54.8; 2, 16.0; 3, 15.7; 4, 13.5
5	102 675	103 280	0.939	−51 254	<0.001	1, 53.0; 2, 15.6; 3, 13.5; 4, 9.1; 5, 8.8
6	102 233	102 960	0.913	−51 016	<0.001	1, 50.6; 2, 15.8; 3, 11.8; 4, 9.0; 5, 7.9; 6, 4.9
7	101 938	102 787	0.922	−50 851	<0.001	1, 51.1; 2, 14.0; 3, 11.4; 4, 8.9; 5, 8.0; 6, 4.9; 7, 1.7
8	101 625	102 597	0.915	−50 678	<0.001	1, 50.5; 2, 10.2; 3, 9.2; 4, 9.1; 5, 8.1; 6, 5.1; 7, 4.2; 8, 3.6
9	101 341	102 434	0.912	−50 518	<0.001	1, 51.8; 2, 9.5; 3, 9.1; 4, 7.4; 5, 6.5; 6, 4.9; 7, 3.7; 8, 3.7; 9, 3.4
10	101 051	102 267	0.916	−50 357	<0.001	1, 50.6; 2, 9.3; 3, 8.9; 4, 7.7; 5, 7.2; 6, 3.9; 7, 3.7; 8, 3.5; 9, 3.1; 10, 2.1

Abbreviations: AIC, Akaike information criterion; BIC, bayesian information criterion; LRT, likelihood ratio test.

### Pediatric Patient Profiles (Latent Classes)

**Figure 2.**
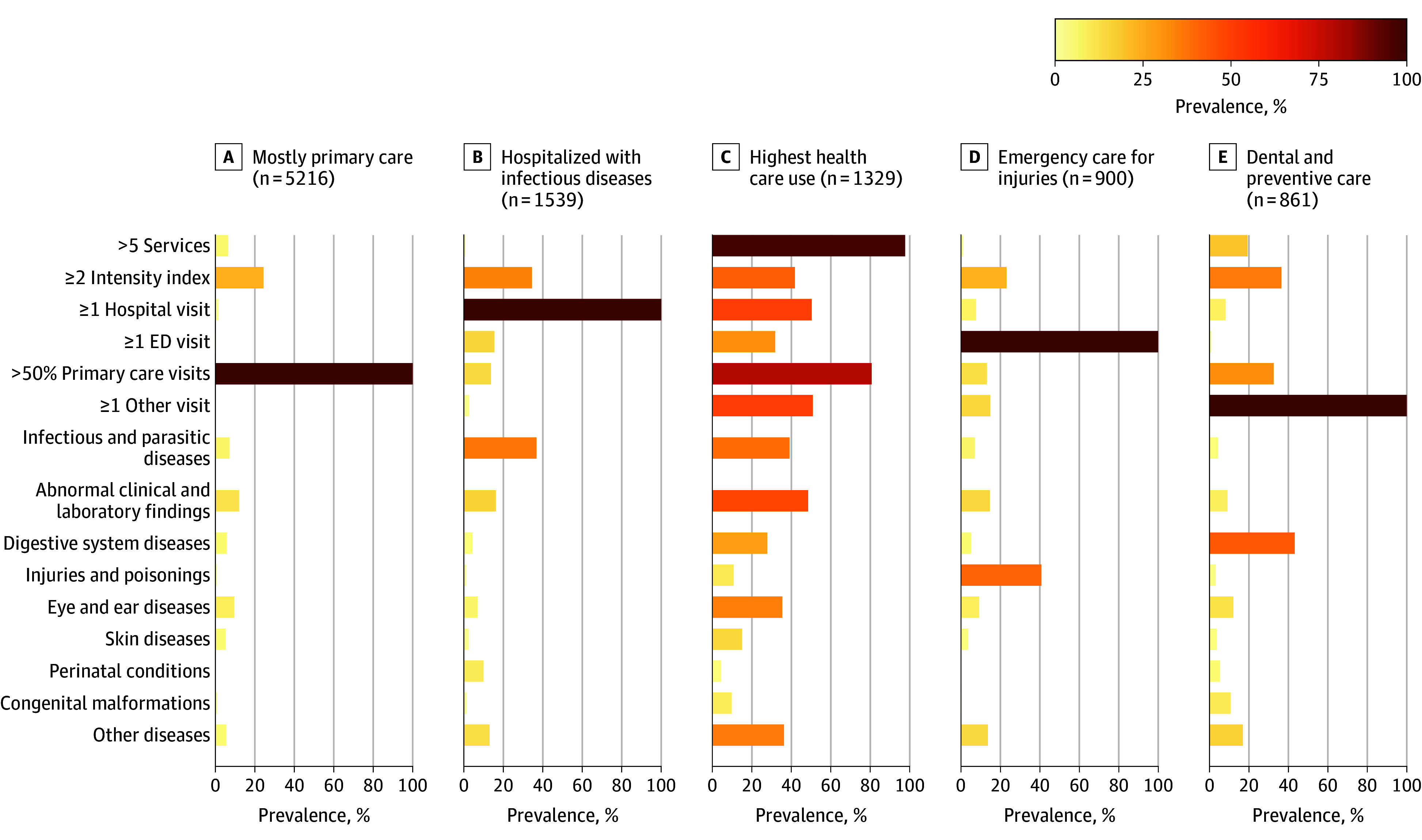
Bar Chart of Prevalence of Health Care Indicators by Latent Class Among Ukrainian Pediatric Patients (n = 9845) ED indicates emergency department.

Based on the latent class analysis, 5 distinct patient profiles were identified, each reflecting unique patterns of diagnoses, health care utilization, and system entry (Figure 2, Table 1; eTable 2 in Supplement 1). The first group, mostly primary care (5216 [53.0%]), involved low system utilization (4156 [79.7%] had 1-3 visits) with exclusive primary care use; later health care system entry; lowest proportion of services received in Kraków; diagnoses mostly involving acute respiratory infections and vaccination administration. The second group, hospitalized with infectious disease*s* (1539 [15.6%]), involved universal hospital use, early system entry, and health care predominantly in Kraków. Common diagnoses were intestinal and acute respiratory infections. The third group, highest health care use (1329 [13.5%]), involved intense health care utilization. Primary care dominated, but many received hospital or outpatient services. Diagnoses were commonly acute respiratory infections or vaccinations and participants experienced the longest median (IQR) stay in system (6 [4-8] months); 715 (53.8%) received services outside of Kraków. The fourth group, emergency care for injuries (900 [9.1%]), involved slightly older patients presenting to the ED with common injury-related diagnoses. Patients typically received 1 service; had early system entry and received care predominantly in Kraków (650 [72.2%]). The fifth group, dental and preventive care (861 [8.7%]), found dental and outpatient specialist care were common. Diagnoses included tooth decay, vaccinations, and acute inflammation of the nose and throat. Later system entry and a median (IQR) stay of 2 (1-4) months were common (Table 1).

Visits for rehabilitation and psychiatric and oncologic diagnoses accounted for 846 encounters (2.4%). Distributions of selected *ICD-10* diagnostic categories across latent health care utilization profiles and the 6 most frequent diagnoses within each latent patient profile are presented in eTables 2 and 3 in Supplement 1, respectively.

### Temporal Patterns of Health Care Use Per Patient Profile

**Figure 3.**
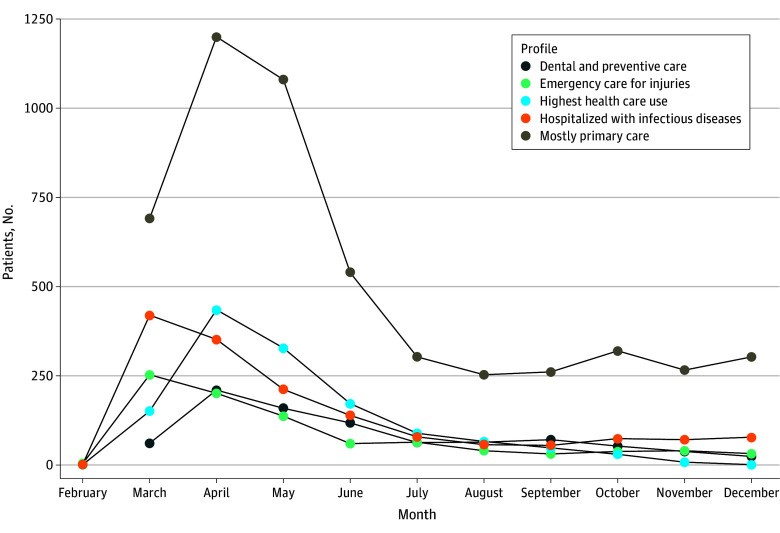
Line Graph of Monthly New Pediatric Ukrainian Refugee Patients Joining Health Care System by Latent Class (n = 9845)

The temporal dynamics of health care system entry revealed distinct patterns reflecting the urgency and nature of health care needs (Figure 3). In March 2022, the hospitalized with infectious diseases and emergency care for injurie*s* profiles peaked first. In April, the mostly primary care, highest health care use, and dental and preventive care profiles reached their peaks. From July onward, new patient enrollments declined across all profiles with the mostly primary care group representing the majority of new entries throughout the remainder of 2022.

Service utilization sequences also differed substantially by profile (eTable 1 in Supplement 1). Patients in the mostly primary care group predominantly had single (2427 [46.5%]) or 3 consecutive primary care visits (1690 [32.4%]). The group hospitalized with infectious diseases typically had single hospital visits (703 [45.7%]) with fewer transitioning to primary care (124 [8.1%]) or presenting with ED-to-hospital pathways (114 [7.4%]). Patients with the highest health care use showed recurrent primary care engagement, while 80 (6.0%) began with hospitalization. Emergency care for injuries patients predominantly had isolated ED visits (486 [54.0%]) with minimal subsequent care. Dental and preventive care users mostly accessed single other visits (186 [21.6%]) or primary care visits (118 [13.7%]).

## Discussion

This cohort study profiled the short-term health care needs of the youngest forced migrant children from Ukraine in southern Poland. Based on data from 9845 patients, 5 distinct patient profiles were identified. Immediately following the invasion, the hospitalized with infectious diseases and emergency care for injuries patient profiles were most common, indicating that young refugee children with acute care needs requiring hospital attention were the focus for the health care system. The following month, more planned health care–seeking behavior was noted among the patients joining the system, with mostly primary care, highest health care use, and dental and preventive care profiles being common. During the latter half of 2022, the mostly primary care group was dominant, indicating a shift toward routine health care services.

The health care system was initially used by patients who sought emergency care, predominantly for injuries, or required hospitalization, largely for gastrointestinal infectious diseases. The difficult circumstances during transit to Poland, including frequent relocation, inadequate sanitation, and residence in group facilities, may have contributed to these observations.^7,19,29^ The findings correlate with other studies on pediatric refugees, which show high ED utilization,^30,31^ as well as respiratory and gastrointestinal illnesses being common reasons for system contact in host countries.^9,13,32^ This study demonstrated that over time, health care utilization patterns shifted toward primary care, which was in line with other studies that indicated decreased specialist and emergency service use once refugees were better connected to care.^33,34^ The finding that certain services, like psychiatry, had low utilization may reflect postdisplacement access barriers or the use of services offered by private, international, or nongovernmental sectors.^35^

It is noteworthy that the health care system serves as a significant point of contact for refugees. This study showed that 9845 refugee patients received services in 2022 at a time when 10 573 children of that age were registered in the area.^3^ Although transient populations may have accessed the system and registered children migrated out of the region due to population movement, likely, a large proportion of refugees connected with the system. As such, the health care system plays a critical role in bridging with pediatric refugees and their families. Social services may be colocated with health care facilities, offering programs aimed at connecting newly arrived families to community resources, increasing health literacy, and taking up public health interventions.^36,37,38^ In Poland, this is especially important as vaccination rates among Ukrainian refugees are lower than among the host population.^39^

It is paramount to meet the health care needs of forced migrant children by having a clearer understanding of their health status.^8^ The process of profiling this population to inform resource allocation is challenging due to its high mobility, which impacts on establishing health care.^36^ Moreover, children fleeing conflict often have lacking or incomplete medical documentation and vaccination histories.^36^ Despite the literature showing that over half of refugee children require additional medical follow-up postarrival,^11,39^ they are at risk for discontinuity of medical care due to frequent relocations and poor medical record transfer.^36^ Information from population-based repositories covering the full spectrum of health services, such as in this study, are needed to fully reflect the health of refugee populations. The use of such data are in line with previous recommendations that advocate for information on refugee children to be systematically collected, such as through repositories or entry assessments,^33,40,41^ to prepare for the unique needs of this population.

### Limitations

This study has some limitations. To date, studies have not statistically profiled young refugee patients using population-based registry data to determine health care needs. Thus, this study’s strength lies in its use of National Health Fund data to comprehensively capture all publicly funded medical visits. Refugee populations are highly mobile and as such, data on patients who moved out of the region would not have been captured. Consequently, longitudinal health care utilization trajectories may have been influenced by population mobility and uncertainty in the underlying denominator population. Nonetheless, this cohort of over 9000 patients, aged 0 to 5 years, represents one of the largest samples examining the health needs of young child refugees in the published literature,^13^ reinforcing the findings. Furthermore, the analysis did not include services offered by private, international, and nongovernmental sectors. The existing databases do not allow for a precise estimation of their share of health care offerings. Based on an earlier analysis, private clinic visits for primary and outpatient care represented a small proportion of all health care visits among Ukrainian refugees,^5^ with emergency and hospital services in the region largely provided by the public system. Additionally, the main *ICD-10* diagnostic code assigned to each health care visit was analyzed, which may not fully capture comorbidities or complex health care encounters. The goal of this analysis was to identify the latent classes of patients based on the chief reason for their health care visits.

## Conclusions

In this cohort study of young Ukrainian refugees in Poland, we identified 5 distinct health care profiles using population-based data. Most children had the primary care services only profile, although more than one-quarter had the hospitalized with infecious diseases and highest health care use profiles. These profiles are valuable to better prepare health systems in host countries to care for pediatric patients who are refugees with specific presentations and care requirements, as well as to prioritize health care needs following crises. These results have important implications for refugee-hosting countries for system readiness and adaptation, indicating that a priority for displaced populations is access to emergency care and inpatient services. Over time, health care utilization patterns reverted to preventive and primary care, with health care utilization decreasing. During the initial months following crises, it may be critical to shift resources to hospital-based settings to meet the medical needs of young refugee children. Future research may examine the health care profiles of older refugee populations, as well as assess patterns in other jurisdictions hosting refugees.

## Notes

Supplement 1. eTable 1. Dominant temporal patterns of healthcare utilization by latent class among pediatric patients aged 0–5 years in the Małopolska Voivodeship, Poland (n = 9,845) eTable 2. Distribution of selected *ICD-10* diagnostic categories across latent healthcare utilization profiles (35,199 services) eTable 3. Top six most frequent diagnoses by latent patient profile (n = 9,845) eFigure 1. Pairwise Jaccard similarity between clinical and utilization indicators among pediatric Ukrainian refugee patients, aged 0 to 5 years, in the Małopolska Voivodeship, Poland (n = 9,845) eFigure 2. Model fit statistics across latent class solutions (AIC, BIC, log-likelihood, and entropy) eFigure 3. Stability of the latent class solution assessed by subsampling (100 subsamples with 80% of the dataset each)

Supplement 2. Data Sharing Statement
