# Supplementary material for: Health Care Utilization Profiles in Young Ukrainian Refugee Children
Source: JAMA Netw Open. 2026 Jul 29;9(7):e2626021. doi: 10.1001/jamanetworkopen.2026.26021 (PMC13421248; doi:10.1001/jamanetworkopen.2026.26021)
Supplement: Supplement 1. — eTable 1. Dominant temporal patterns of healthcare utilization by latent class among pediatric patients aged 0–5 years in the Małopolska Voivodeship, Poland (n = 9,845) eTable 2. Distribution of selected ICD-10 diagnostic categories across latent healthcare utilization profiles (35,199 services) eTable 3. Top six most frequent diagnoses by latent patient profile (n = 9,845) eFigure 1. Pairwise Jaccard similarity between clinical and utilization indicators among pediatric Ukrainian refugee patients, aged 0 to 5 years, in the Małopolska Voivodeship, Poland (n = 9,845) eFigure 2. Model fit statistics across latent class solutions (AIC, BIC, log-likelihood, and entropy) eFigure 3. Stability of the latent class solution assessed by subsampling (100 subsamples with 80% of the dataset each) [file jamanetwopen-e2626021-s001.pdf]

Supplemental Online Content

Dobies B, Pędziwiatr K, Bielska IA. Health care utilization profiles in young Ukrainian refugee children. *JAMA Netw Open.* 2026;9(7):e2626021.
doi:10.1001/jamanetworkopen.2026.26021

eTable 1. Dominant temporal patterns of healthcare utilization by latent class among pediatric patients aged 0–5 years in the Małopolska Voivodeship, Poland (n = 9,845)

eTable 2. Distribution of selected *ICD-10* diagnostic categories across latent healthcare utilization profiles (35,199 services)

eTable 3. Top six most frequent diagnoses by latent patient profile (n = 9,845)

eFigure 1. Pairwise Jaccard similarity between clinical and utilization indicators among pediatric Ukrainian refugee patients, aged 0 to 5 years old, in the Małopolska Voivodeship, Poland (n = 9,845)

eFigure 2. Model fit statistics across latent class solutions (AIC, BIC, log-likelihood, and entropy)

eFigure 3. Stability of the latent class solution assessed by subsampling (100 subsamples with 80% of the dataset each)

This supplemental material has been provided by the authors to give readers additional information about their work.

© 2026 Dobies B et al. *JAMA Network Open*.

eTable 1. Dominant temporal patterns of healthcare utilization by latent class among pediatric patients aged 0–5 years in the Małopolska Voivodeship, Poland (n = 9,845).

Profile	Trajectory (Visit 1 → Visit 2 → Visit 3)	n (%)
Mostly primary care	PC → None → None<br>PC → PC → PC<br>PC → PC → None	2,427 (46.5)<br>1,690 (32.4)<br>1,076 (20.6)
Hospitalized with infectious diseases	Hospital → None → None<br>Hospital → PC → PC<br>ER→ Hospital → None	703 (45.7)<br>124 (8.1)<br>114 (7.4)
Highest healthcare use	PC → PC → PC<br>Hospital → PC → PC<br>PC → PC → Other	619 (46.6)<br>80 (6.0)<br>69 (5.2)
Emergency care for injuries	ER → None → None<br>ER → PC → PC<br>ER → PC → None	486 (54.0)<br>53 (5.9)<br>50 (5.6)
Dental and preventive care	Other → None → None<br>PC → PC → PC<br>PC → PC → Other	186 (21.6)<br>118 (13.7)<br>88 (10.2)

PC, primary care; ER, emergency room

© 2026 Dobies B et al. *JAMA Network Open*.

eTable 2. Distribution of selected ICD-10 diagnostic categories across latent healthcare utilization profiles (35,199 services).

ICD-10 diagnostic category	**Latent class,** no. of services				
	**Highest healthcare use,** n = 14,293	**Mostly primary care,** n = 12,358	**Dental and preventive care,** n = 3,451	**Emergency care for injuries,** n = 1,842	**Hospitalized with infectious diseases,** n = 3,255
Intestinal infectious (A00-A09)	292 (2.0%)	80 (0.6%)	<5 (<0.1%)	32 (1.7%)	**562 (17.3%)**
Neoplasms and cancer treatment (C00-D48 & Z51.0-Z51.3)	**179 (1.3%)**	11 (0.1%)	24 (0.7%)	0 (0%)	7 (0.2%)
Respiratory (J00-J99)	4,743 (33.2%)	**5,702 (46.1%)**	661 (19.2%)	467 (25.4%)	980 (30.1%)
Oral cavity, salivary glands and jaws (K00-K14)	424 (3.0%)	95 (0.8%)	**661 (19.2%)**	33 (1.8%)	10 (0.3%)
Injury (S00-T98)	409 (2.9%)	76 (0.6%)	67 (1.9%)	**610 (33.1%)**	40 (1.2%)
Vaccination received/planned (Z23-Z27)	1,034 (7.2%)	**1,656 (13.4%)**	272 (7.9%)	50 (2.7%)	66 (2.0%)

Note: Bolded values indicate the highest proportion (%) within each ICD-10 diagnostic category across the latent healthcare utilization profiles.

eTable 3. Top six most frequent diagnoses by latent patient profile (n = 9,845).

Top six diagnoses per patient profile	ICD-10 code	Services, n (%)	Patients, n (%)	Age, median (Q1, Q3)
**Mostly primary care** (5,216 patients; 12,358 services)				
Acute nasopharyngitis (common cold)	J00	1754 (14.2)	1357 (26.0)	3 (1, 4)
Acute upper respiratory infections of multiple and unspecified sites	J06	1484 (12.0)	1150 (22.0)	3 (2, 4)
Need for immunization against combinations of infectious diseases	Z27	932 (7.5)	627 (12.0)	1 (0, 1)
Acute pharyngitis	J02	450 (3.6)	398 (7.6)	3 (2, 4)
Acute bronchitis	J20	399 (3.2)	302 (5.8)	3 (2, 4)
Medical observation and evaluation for suspected diseases and conditions ruled out	Z03	337 (2.7)	274 (5.3)	2 (1, 4)
**Hospitalized with infectious diseases** (1,539 patients; 3,255 services)				
Rotaviral enteritis	A08.0	217 (6.7)	185 (12.0)	3 (2, 4)
Acute upper respiratory infections of multiple and unspecified sites	J06	206 (6.3)	173 (11.2)	3 (2, 4)
Acute nasopharyngitis (common cold)	J00	197 (6.1)	169 (11.0)	3 (2, 4)

© 2026 Dobies B et al. *JAMA Network Open*.

Other gastroenteritis and colitis of infectious and unspecified origin	A09	194 (6.0)	151 (9.8)	3 (2, 4)
Lifeborn infants	Z38/Z38.0	156 (4.8)	94 (6.1)	0 (0, 0)
Nausea and vomiting	R11	91 (2.8)	78 (5.1)	3 (2, 4)
**Highest healthcare use** (1,329 patients; 14,293 services)				
Acute nasopharyngitis (common cold)	J00	1195 (8.4)	666 (50.1)	3 (1, 4)
Acute upper respiratory infections of multiple and unspecified sites	J06	1092 (7.6)	586 (44.1)	3 (2, 4)
Need for immunization against combinations of infectious diseases	Z27	592 (4.1)	307 (23.1)	1 (0, 1)
Medical observation and evaluation for suspected diseases and conditions ruled out	Z03	396 (2.8)	280 (21.1)	2 (1, 4)
Acute pharyngitis	J02	365 (2.6)	265 (19.9)	3 (2, 4)
Acute bronchitis	J20	350 (2.4)	207 (15.6)	3 (1, 4)
**Emergency care for injuries** (900 patients; 1,842 services)				
Acute upper respiratory infections of multiple and unspecified sites	J06	128 (6.95)	109 (12.1)	3 (2, 4)
Acute nasopharyngitis (common cold)	J00	113 (6.1)	91 (10.1)	3 (2, 4)
Open wound of head/scalp	S01/S01.0	93 (5.0)	72 (8.0)	4 (3, 4)
Superficial injury of scalp	S00.0	47 (2.6)	43 (4.8)	2 (1, 4)
Procedure not carried out because of patient's decision for other and unspecified reasons	Z53.2	40 (2.2)	40 (4.4)	3 (1, 4)
Nausea and vomiting	R11	36 (2.0)	36 (4.0)	3 (2, 4)
**Dental and preventive care** (861 patients; 3,451 services)				
Dental caries (tooth decay)	K02	411 (11.9)	232 (26.9)	4 (4, 5)
Need for immunization against combinations of infectious diseases	Z27	187 (5.4)	104 (12.1)	1 (0, 1)
Acute nasopharyngitis (common cold)	J00	185 (5.4)	131 (15.2)	3 (1, 4)
Medical observation and evaluation for suspected diseases and conditions ruled out	Z03	121 (3.5)	87 (10.1)	2 (1, 4)
Acute upper respiratory infections of multiple and unspecified sites	J06	119 (3.4)	101 (11.7)	3 (2, 4)
Diseases of pulp and periapical tissues	K04	110 (3.2)	77 (8.9)	5 (4, 5)

Q1, Q3: first and third quartiles

© 2026 Dobies B et al. *JAMA Network Open*.

eFigure 1. Pairwise Jaccard similarity between clinical and utilization indicators among pediatric Ukrainian refugee patients, aged 0 to 5 years old, in the Małopolska Voivodeship, Poland (n = 9,845).

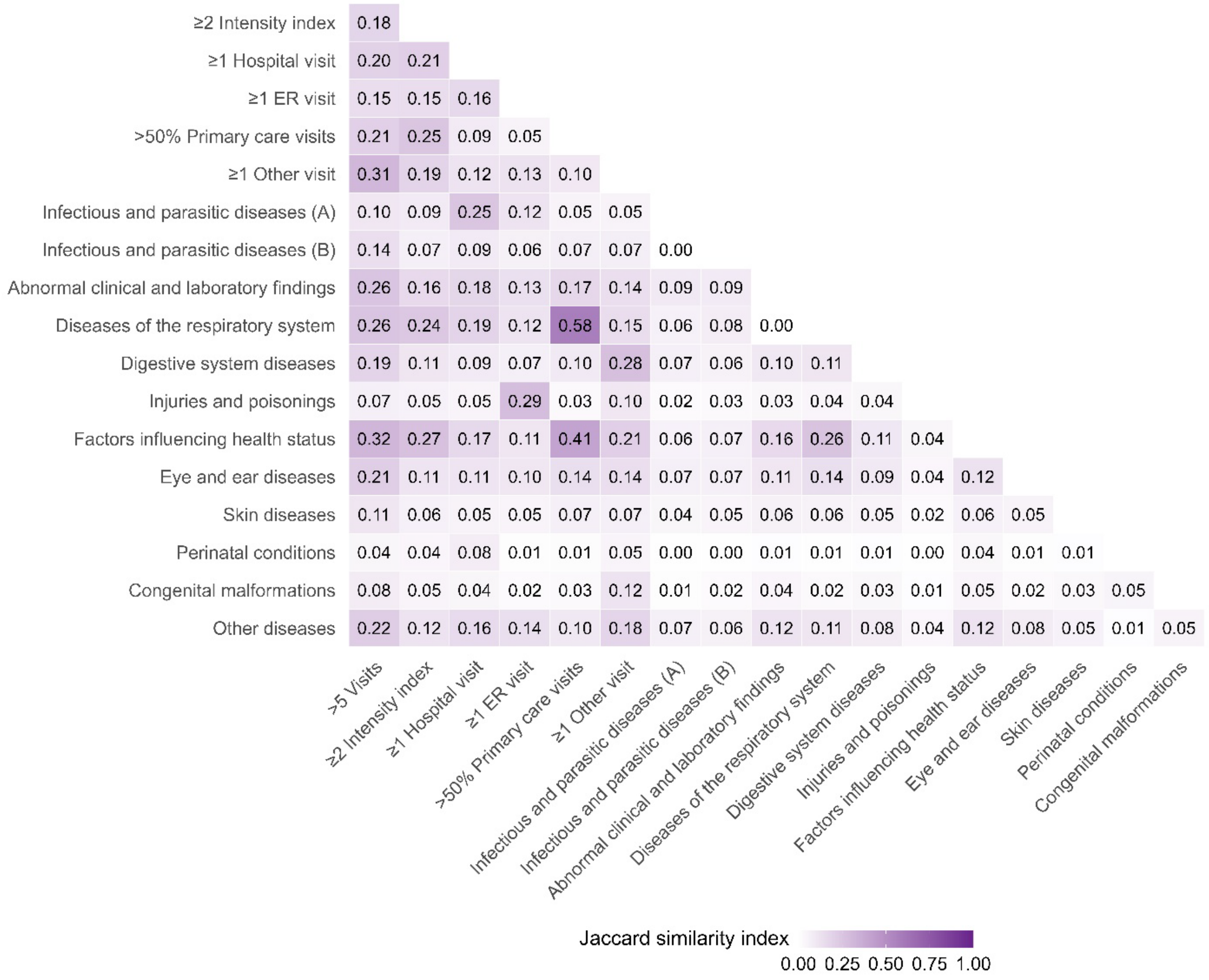

© 2026 Dobies B et al. *JAMA Network Open*.

eFigure 2. Model fit statistics across latent class solutions (AIC, BIC, log-likelihood, and entropy).

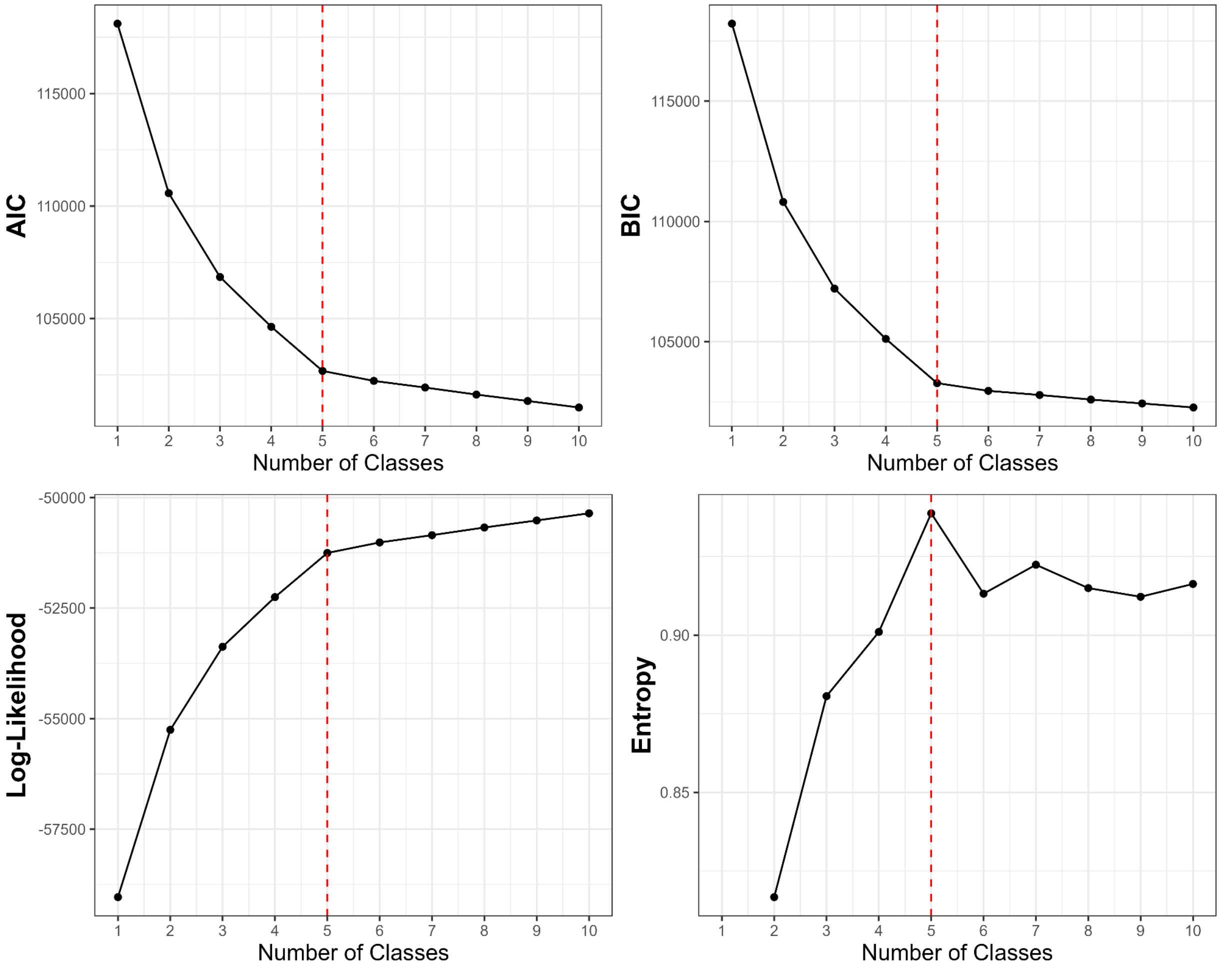

AIC, Akaike Information Criterion; BIC, Bayesian Information Criterion

© 2026 Dobies B et al. *JAMA Network Open*.

eFigure 3. Stability of the latent class solution assessed by subsampling (100 subsamples with 80% of the dataset each)

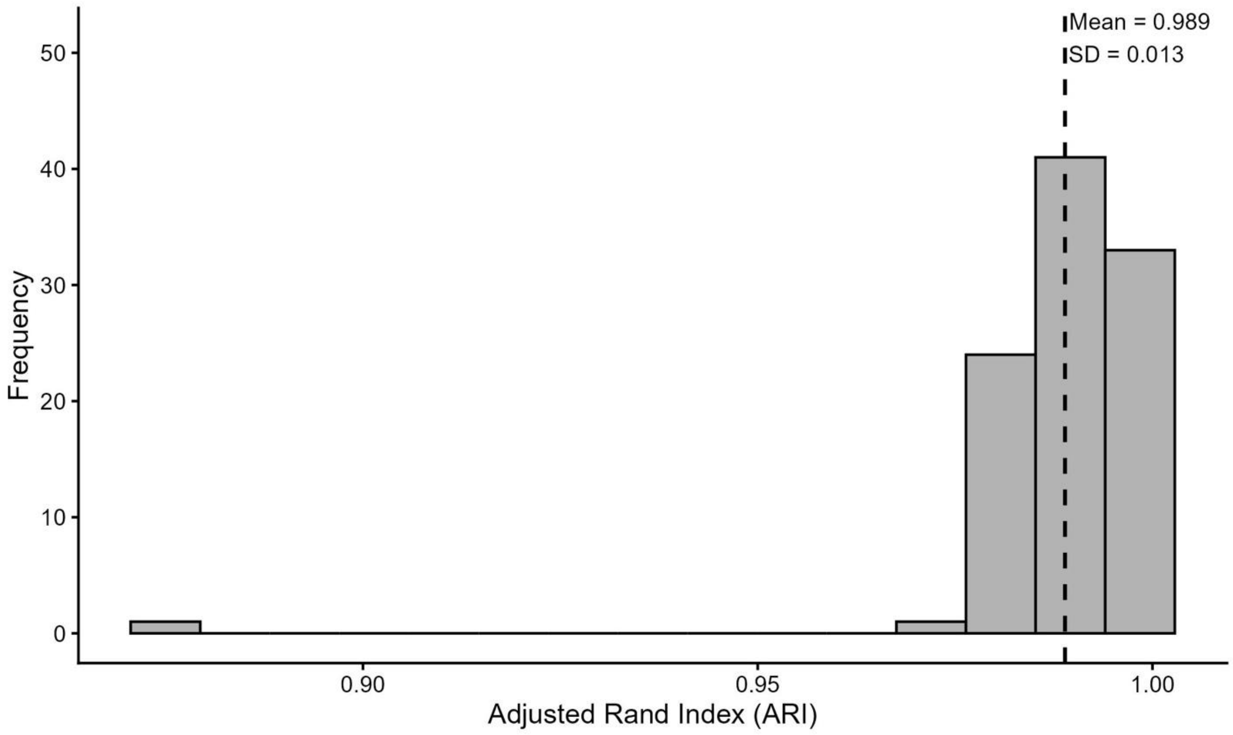

© 2026 Dobies B et al. *JAMA Network Open*.
