# Supplementary material for: Health Care Utilization Profiles in Young Ukrainian Refugee Children
Source: JAMA Netw Open. 2026 Jul 29;9(7):e2626021. doi: 10.1001/jamanetworkopen.2026.26021 (PMC13421248; doi:10.1001/jamanetworkopen.2026.26021)
Supplement: Supplement 2. — Data Sharing Statement [file jamanetwopen-e2626021-s002.pdf]

Data Sharing Statement

Dobies. Health Care Utilization Profiles in Young Ukrainian Refugee Children. *JAMA Netw Open*. Published July 29, 2026. doi:10.1001/jamanetworkopen.2026.26021

Data
Data available: No

Additional Information
Explanation for why data not available: The data analyzed for this study can be requested from the data holder, the National Health Fund in Poland.
